# The use of cone beam computed tomography by brazilian endodontists: A questionnaire-based survey 

**DOI:** 10.4317/jced.60855

**Published:** 2023-11-01

**Authors:** Hermano-Camelo Paiva, Eduardo Akisue, Felipe-Potgornik Ferreira, Karla-Nogueira Matos, Hasan Al Zaibak, Iandara-de Lima Scardini, Celso-Luis Caldeira, George-Táccio-de Miranda Candeiro, Giulio Gavini

**Affiliations:** 1 Department of Restorative Dentistry, School of Dentistry, University of São Paulo, São Paulo, Brazil; 2 Discipline of Endodontics, University Santa Cecilia, Santos, Brazil; 3 Department of Restorative Dentistry, Unichristus University Center, Fortaleza, Brazil

## Abstract

**Background:**

This study aimed to assess the knowledge of Brazilian endodontists regarding the use of cone beam computed tomography (CBCT) in endodontic therapy.

**Material and Methods:**

An invitation to participate in the research was sent to 3256 professionals through class groups on social networks and via email. Participants answered an online questionnaire consisting of 11 questions about the clinical situations in which they used CBCT and whether they had any knowledge of the technical protocols such as parameters of field of view (FOV) and voxels of the CBCT equipment. The questionnaire was answered by 742 endodontists who represented 22.7% of the invited professionals.

**Results:**

The data obtained indicate that 76.7% of the participants use or have used CBCT during endodontic treatments. The most often reported clinical conditions for using the CBCT were: root fractures (64%), locating the canals (58.7%), perforations (53.9%), and root resorption (42.1%). More than 60% of the participants stated that they do not have information about the voxel, FOV, and the CBCT system used in their requests. Only 34.1% reported using CBCT to the performed endodontic treatments follow-up.

**Conclusions:**

CBCT was a tool widely utilized by the participants, with root fracture being the clinical condition with the highest indication for CBCT. Many professionals were unaware of the technical protocol used in the exams.

** Key words:**Endodontics, Cone Beam Computed Tomography, Endodontic Therapy.

## Introduction

Cone Beam Computed Tomography (CBCT) is an imaging method introduced into dentistry in 1998 (1). There has been a growing interest in its applications, and its use has spread widely in different dental specialties, such as oral and maxillofacial surgery, orthodontics, and endodontics (2,3).

CBCT allows for the three-dimensional visualization of one or more teeth and their relation with adjacent tissues, offering higher sensitivity, specificity, and accuracy compared to periapical radiographs (4). Thus, CBCT has been considered a reliable tool to aid in the diagnosis, promoting substantial impact on decision making and planning, especially in cases of high difficulty (5-7).

In endodontics, CBCT is a valuable assisting tool for the diagnosis of apical periodontitis (8,9), vertical and horizontal root fractures (10,11), perforations, and dental resorption (12), locating calcified canals (13), planning endodontic surgery (14), and dental auto-transplantation (15). Furthermore, CBCT has been employed in endodontic treatments. Studies show that CBCT has higher accuracy than electronic apex locators for determining the working length (16-18). Other papers demonstrate the auxiliary use of CBCT in constructing guides for more accurate and conservative approaches, especially in cases involving calcified canals (19).

The knowledge of ideal technical protocol for CBCT helps in reducing radiation exposure to patients, following the ALARA (as low as reasonably achievable) principle (20). CBCT examination should only be requested by a clinician who has adequate knowledge of its endodontic applications, who has experience in the interpretation of images, and who appreciates the limitations of CBCT (20). Therefore, to enhance the use of CBCT, the specialist needs to know the advantages and indications for utilizing it as an auxiliary tool in endodontic diagnosis, choosing the most suiTable equipment according to the evaluated structure, and interpreting tomographic images.

The use of CBCT imaging in private endodontic practice in Brazil is unexplored. No studies have been carried out yet to examine the experience or adoption of CBCT use by Brazilian endodontists. This study investigates the adoption, accessibility, knowledge, and use of CBCT imaging among Brazilian specialists.

## Material and Methods

The study was carried out in accordance with ethical principles and guidelines for research involving human beings and was exempted from consideration by the Ethics Committee in accordance with resolution nº 510, of april 7, 2016 of the national health council.

3256 dentists received an invitation to participate in the online questionnaire via google forms links, from social network groups, and by email. The invitation informed the participants that the anonymous online survey is part of a research project in a Brazilian public university. The surveys filled a questionnaire with questions from 01 to 11, relying on individual responses. Partakers, who were not specialists or not pursuing a specialty in endodontics, were excluded.

**Figure 1:**
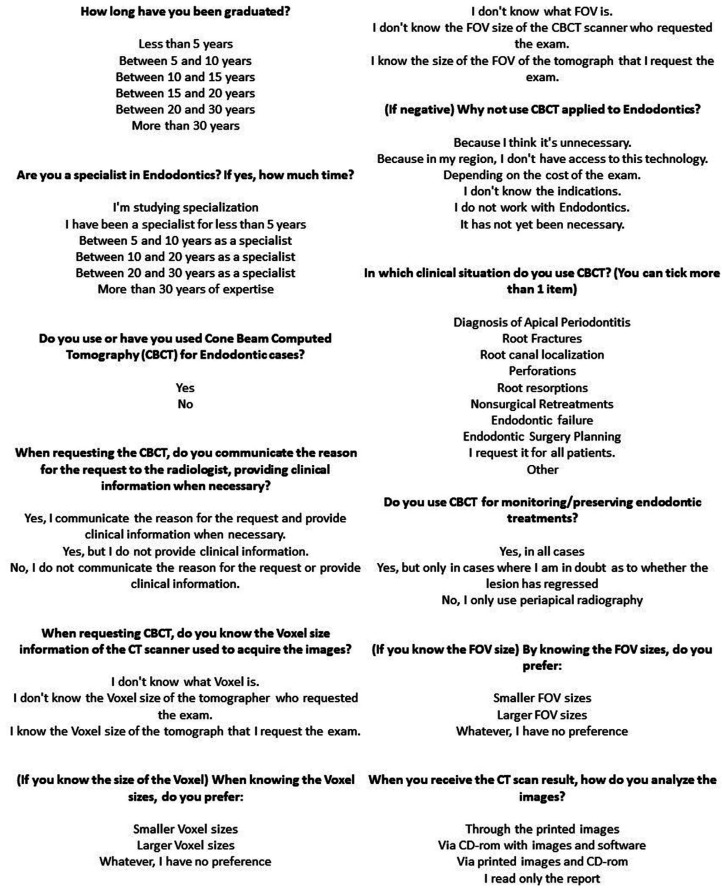
A survey of CBCT use among Brazilian specialists in endodontics (Summary questionnaire).

The first part of the questionnaire corresponds with the dentist’s qualification and the use of CBCT in endodontic cases. The participants, who stated that they don’t use CBCT, were asked about the non-use in their clinical practice. For those who utilize the technology, the questionnaire provided additional questions, obtaining further details; Such as the clinical condition for use, the form for the request, preferred FOV, voxel size, and the type of analysis of the acquired exam. A copy of the questionnaire sent to the participants is available in Figure 1.

The collected data were analyzed using Jamovi software and descriptive statistics, presented as absolute values (n) and percentages (%). For the statistical analysis involving the length of professional experience, chi-square and Fisher’s exact tests were used, considering a significance level of *p*<0.05.

## Results

742 professionals answered the questionnaire, representing 22.7% of the invited dentists. Regarding the specialty in endodontics, 210 (28%) were still in training, 349 (47%) had been specialists for less than ten years, and 183 (25%) had been specialists for more than ten years.

**Figure 2:**
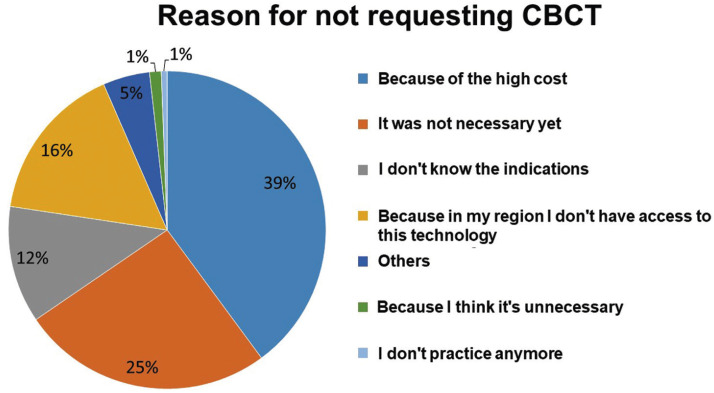
Reason why survey participants do not request CBCT.

**Figure 3:**
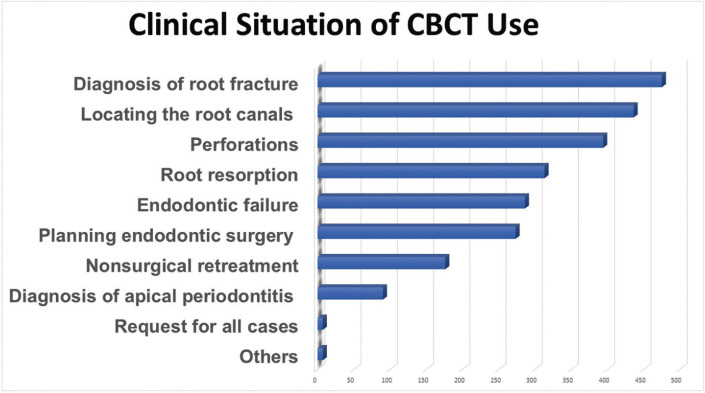
Bar chart of clinical situation in which participants most often request a CBCT.

The data revealed that 76.7% of the participants reported using or having used CBCT in endodontic therapy at some point. The length of professional experience is significantly associated with the use of CBCT, with more experienced professionals being the ones who use it the most (*p*<0.05) (Table 1). Among those who do not utilize it, 39.8% responded that it was due to the cost of the exam, and 25.6% stated that it was not necessary. Figures 2 and 3 presents the reason for not request CBCT and clinical conditions when CBCT is most requested, respectively.

**Table 1:**
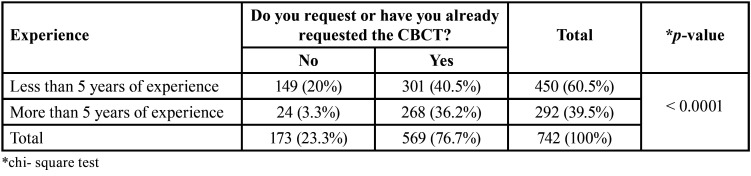
Association between time of experience and use of CBCT.

Regarding cases follow-up, 35% of the participants claimed that they use CBCT for the procedure, and 65% only use periapical x-rays.

Among the participants that solicit a CBCT, 82% said that they communicate with the radiologist and provide clinical information when necessary, 15% inform the radiologist regarding the reason for the request, and 3% do not tell the reason for the request.

For 44% of the respondents, they are not familiar with the used scanner in their requests. 31% use Prexxion, 13.3% use I-Cat, 4.4% use 3D Accuitomo, and 7.3% use other CT scanners.

75% and 64% of the surveys confirmed that they do not know about FOV and Voxel, respectively, of the requested CBCT images. Among those who knew, 90% said they prefer a smaller FOV, 5% larger FOV, and 5% did not have any preference. Moreover, 91.7% informed they favor a smaller voxel, 5% larger voxel, and 3.3% did not prefer the size.

For image evaluation, 75% of the participants claimed using some software, and 25% claimed to examine only printed images. There was no significant association between length of professional experience and the mode of tomographic images analysis (*p*>0.05).

**Figure 4:**
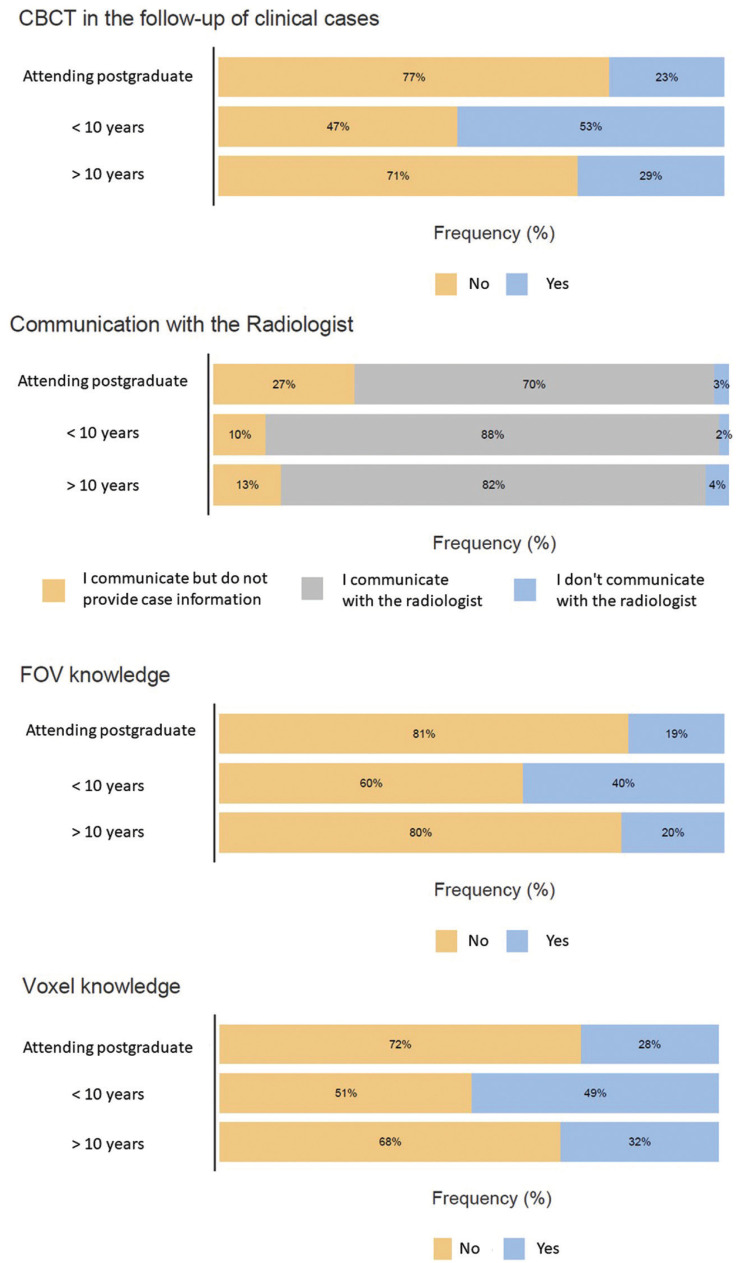
Stacked bar of the association between clinical experience and CBCT in the follow-up of clinical cases, Communication with the radiologist, FOV knowledge and voxel knowledge.

The length of professional experience is significantly associated with its use in the follow-up of the cases, the type of request/communication with the radiological center, and the knowledge about the FOV and Voxel of the scanner used, with the most experienced professionals being the ones who know the most in this regard (*p*<0.05) (Fig. 4).

Only 177 (31%) of the 571 professionals who claimed that they use CBCT in Endodontics have adequate knowledge about the technical protocol of requesting and analyzing the images. In other words, they are familiar with the FOV and voxel of the scanner and favor the smaller ones, communicate with the radiologist about the reason for the imaging request, and do not only use the report to analyze the exams. The length of the professional experience is significantly associated with adequate knowledge about how to request and analyze CBCT images. Professionals with higher experience had more knowledge (*p*<0.05).

## Discussion

The study gathered information on the knowledge of endodontists and professionals undertaking a specialty in endodontics regarding the request and application of CBCT in endodontic therapy. Participants in this study were invited through social networks and email, obtaining 742 responses. The total number of Brazilian specialists is 14,235 between active and non-active professionals, according to a survey carried out in January 2022 on the official website of the Federal Council of Dentistry. The respondents represent 5.2% of the total number of specialists in Brazil. For the variables discussed here, we can accept that the distribution of the present sample represents the active population of specialists.

CBCT is currently considered a widely used important asset in endodontic therapy. It can aid in locating the canals, pre-surgical examination, evaluating the quality of treatments, in addition to the diagnosis of root fractures, resorption, perforations, and apical periodontitis (6,20,21). Furthermore, this technology is critical for the decision-making of specialists, especially in cases of high complexity (22,25). Many studies evaluated the adoption, application, and analysis of CBCT in dentistry based on surveys and questionnaires, providing descriptive analyses of the results (3,7,23).

The present study shows that the majority of respondents (more than 70%) use or have used CBCT in endodontic therapy (Table 1). Few studies have evaluated the popularity of CBCT since the beginning of the increasing adoption of this technology. A previous study conducted in Turkey reported that 41.9% of dentists had previously referred their patients for a CBCT (24), which is lower than the percentage reported in the present study. A similar investigation in American endodontists showed that 91.8% use CBCT in clinical practice (25).

The clinical conditions in which the participants most requested CBCT were to aid with the diagnosis of root fracture (64%), followed by locating the canals (58.7%), perforations (53.9%), root resorption (42.1%), endodontic failure (38.5%), planning endodontic surgery (36.8%), nonsurgical retreatment (23.7%) and diagnosis of apical periodontitis (12.2%), as shown in Figure 2. Several studies showed the high sensitivity and specificity of CBCT for the diagnosis of root fractures (5,26,27), which proves that most participants know the indications for using CBCT in endodontic therapy.

Only 33% of the participants reported using CBCT for endodontic treatment follow-up, and 15.6% use it for apical periodontitis diagnosis. We know that for the diagnosis of apical periodontitis, CBCT is considered the gold standard since periapical radiographs can display distortions and overlapping of anatomical structures, frequently underestimating the size of periapical bone rarefaction (5). Studies also showed that CBCT is crucial for detecting apical periodontitis, mainly when a new lesion develops after the endodontic treatment, even though the bone rarefaction was absent before the procedure (21).

According to some authors, decision-making for dental treatments may vary between dentists and specialists, depending on the professional’s level of training and clinical experience (7,28,29). Other authors mentioned that the years of professional experience directly influence the diagnostic ability of the dentist, making it more accurate (35-37). In this study, endodontists with more than ten years of experience had more knowledge about the size of FOV and voxel used (31%).

Currently, various CBCT systems are used in dental practice, with different sizes of FOV and voxel, depending on the clinical indication. It is essential to know the FOV and voxel of the scanner employed, as they directly influence the quality of images and visualization of target structures in endodontics, with a preference to smaller ones (30), as well as exposing the patient to lower doses of radiation, following the ALARA principle (32). Most of the participants in this study who request or have already requested CBCT are not familiar or do not know the size of the FOV (74.8%) and voxel (65%) of the CT scanners utilized.

The information gathered from a CBCT exam can influence the treatment plan, leading to alterations in about 26% to 27% of the cases (7,31). In this study, more than 40% of professionals use printed images and CDs to assess the tomographic exam; however, the practitioner needs to be regularly updated, incorporating new tomographic examination tools, such as dynamic visualization software, which help in the diagnosis, planning, and treatment of complex endodontic cases (33).

The present study demonstrates that only 31% of the participants have sufficient knowledge of the technical protocol that best improves the use of CBCT in endodontic therapy. In other words, they have experience with the FOV and voxel of the scanner used and prefer the smaller ones, relate to the radiologist the reason for the clinical request, and do not only use the report to analyze the exams. Although CBCT is advantageous when requesting complementary imaging exams, the professional must consider the ALARA principle. Discussing the benefits and potential risks with the patient in advance is a must, and even with the relatively low effective dose, CBCT should be appropriately used (32).

## Conclusions

The study concludes that CBCT is a widely used tool among the participating dentists, with the highest indication for root fractures of the clinical conditions. We observed that many professionals do not have information regarding the technical protocol used in CBCT exams.
